# An assessment of the supply, programmatic use, and regulatory issues of single low-dose primaquine as a *Plasmodium falciparum* gametocytocide for sub-Saharan Africa

**DOI:** 10.1186/s12936-015-0714-3

**Published:** 2015-05-15

**Authors:** Ingrid Chen, Eugenie Poirot, Mark Newman, Deepika Kandula, Renee Shah, Jimee Hwang, Justin M. Cohen, Roly Gosling, Luke Rooney

**Affiliations:** Global Health Sciences, University of California, San Francisco, 550 16th Street, 3rd Floor, San Francisco, CA 94158 USA; Clinton Health Access Initiative, Boston, MA USA; Malaria Branch, Centers for Disease Control and Prevention, 1600 Clifton Rd, Atlanta, GA USA

**Keywords:** Primaquine, 8-aminoquinoline, *Plasmodium falciparum*, Africa, Glucose-6-phosphate dehydrogenase deficiency, G6PD, Drug dosing, Drug production, Drug manufacturing, Drug regulation, Malaria transmission

## Abstract

**Background:**

Global ambitions to eliminate malaria are intensifying, underscoring a critical need for transmission blocking tools. In 2012, the WHO recommended the use of 0.25 mg/kg of single low-dose (SLD) primaquine to stop *Plasmodium falciparum* transmission. To ensure the availability of SLD primaquine to countries in need of this tool, more information on the supply, programmatic, and regulatory barriers to the rollout of SLD primaquine is required.

**Methods:**

Challenges to the rollout of SLD primaquine in sub-Saharan Africa were established through semi-structured qualitative interviews with three primaquine manufacturers, 43 key informants from Ethiopia, Senegal, Swaziland, Zambia, and Tanzania, and 16 malaria research experts.

**Results:**

Sanofi and Remedica are the only two sources of SRA-approved primaquine suitable for procurement by international donors. Neither manufacturer produces primaquine tablet strengths suitable for the transmission blocking indication.

In-country key informants revealed that the WHO weight-based recommendation to use SLD primaquine is challenging to implement in actual field settings. Malaria programmes expressed safety concerns of SLD primaquine use in individuals with glucose-6-phosphate dehydrogenase (G6PD) deficiency, as well as potential interactions between primaquine and co-morbidities, and drug-drug interactions with HIV and/or tuberculosis treatments. Regulatory processes are a major barrier to the rollout of SLD primaquine, requiring multiple steps at both the country and global level. Despite these barriers, demand for SLD primaquine is growing, and malaria researchers are interested in primaquine deployment through mass screen and treat and/or mass drug administration campaigns.

**Conclusion:**

Demand for primaquine as a transmission blocking agent is growing rapidly yet multiple barriers to SLD primaquine use exist. Research is needed to define the therapeutic dose range, which will guide dosing regimens in the field, inform the development of new, lower strength primaquine tablets and/or formulation(s), and allay programmatic safety concerns in individuals with G6PD deficiency. Potential interactions between primaquine and co-morbidities and treatments should be explored. To minimize regulatory delays, countries need to prepare for product registration at an early stage, WHO prequalification for suitable primaquine tablet strengths and/or new formulations should be sought, and in the meanwhile only Stringent Regulatory Authority (SRA)-approved primaquine should be used.

**Electronic supplementary material:**

The online version of this article (doi:10.1186/s12936-015-0714-3) contains supplementary material, which is available to authorized users.

## Background

Over the past 13 years, the success of malaria control programmes have contributed to a decrease in malaria mortality rates by approximately 50% worldwide [1]. To maintain these gains, the World Health Organization (WHO) recommends use of the drug primaquine, in conjunction with an artemisinin-based combination therapy (ACT), to block *Plasmodium falciparum* transmission in areas approaching malaria elimination and/or facing artemisinin resistance [2].

Primaquine is an old drug that has been on the market for over 60 years [3]. Originally developed as a 14-day course of treatment for the radical cure of *Plasmodium vivax* malaria, there is now growing interest in a second indication for primaquine, as a single low dose, to clear mature, late-stage *P. falciparum* gametocytes. As primaquine is the only commonly used anti-malarial on the market that kills mature gametocytes, the malaria life cycle stage responsible for the transmission of malaria from the human to the mosquito, its use for this second indication is expected to accelerate efforts to eliminate *P. falciparum* malaria [4].

The use of primaquine is often hampered by safety concerns, as it is known to cause dose-dependent haemolysis in individuals deficient in glucose-6-phosphate dehydrogenase (G6PD), an enzyme involved in the pentose phosphate pathway [5]. Haemolytic side-effects depend mostly on the dose of primaquine used and the host variant of G6PD deficiency, and can range anywhere from asymptomatic haemolysis to acute haemolytic anaemia, and in rare cases, renal failure and death [5]. While the 14-day course of primaquine (0.25–0.5 mg/kg per day for 14 days) treatment required for the radical cure of *P. vivax* malaria is considered to be unsafe for G6PD-deficient (G6PDd) individuals, the WHO considers a single low dose (0.25 mg/kg) of primaquine to be safe for individuals with G6PD-deficiency, as implied by their recommendation to use primaquine as a *P. falciparum* gametocytocide [6].

The WHO recommendation for single low-dose (SLD) primaquine use was issued in October 2012, and does not come with the statement “Strong recommendation, high quality evidence” that typically accompanies WHO recommendations for anti-malarial use [6]. The recommendation was based on a pooled analysis of unstandardized infectivity studies, and the dose was lowered from the previously recommended 0.75 mg/kg dose, excluding G6PDd individuals, uptake of which was limited due to safety concerns and a lack of infrastructure for G6PD testing [2]. Since 2012, three countries in the Greater Mekong Subregion have adopted SLD primaquine as policy, and seven countries in sub-Saharan Africa have written SLD primaquine into policy documents, although the actual level of implementation and adherence to these policies is unclear [7].

Since SLD primaquine is recommended by the WHO and is being written into country policies, its use as a *P. falciparum* gametocytocide is expected to grow. However, the global production and supply of primaquine are still tailored for its use for *P. vivax* malaria, at different dose regimens, and hence with a different safety profile for G6PDd individuals. The label for primaquine lists its indication for the radical cure of *P. vivax* malaria only; dose instructions are for *P. vivax* malaria, and the use of primaquine for *P. falciparum* remains off-label. As the use of SLD primaquine for *P. falciparum* malaria grows, changes in the global demand for primaquine are expected and changes in the global supply will thus be required.

This study was conducted to identify supply, programmatic, and regulatory barriers to the rollout of SLD primaquine for *P. falciparum* elimination in sub-Saharan Africa, where *P. falciparum* comprises the vast majority of malaria infections. The study was a research priority in the SLD primaquine roadmap, devised from a meeting of stakeholders in 2012 to facilitate the deployment of SLD primaquine in sub-Saharan Africa [8]. This paper is a summary of the key findings from a full report that can be found elsewhere [9].

## Methods

### Manufacturers

In order to explore the production landscape of primaquine and market demand for SLD primaquine, select manufacturers of primaquine were interviewed (interview guide in Additional file 1). Manufacturers of primaquine were identified through a review of procurement databases, life sciences analyst reports, drug regulatory agency databases, drug company websites, and scientific and public health reports. From a total of 51 manufacturers of primaquine identified, two manufacturers that produce Stringent Regulatory Authority (SRA)-approved primaquine, Sanofi and Remedica, and the main manufacturer of the active pharmaceutical ingredient (API) of primaquine, Ipca, were selected for interview. Sanofi and Remedica, the only producers of SRA-approved primaquine, were interviewed because international donors, such as the Global Fund to fight AIDS, Tuberculosis and Malaria (GFATM), require that either SRA approval or WHO prequalification be obtained for all drugs to be procured through their funding mechanism, and there is no WHO prequalified primaquine product.

As defined by the WHO, a SRA is a regulatory authority which is: (a) a member of the International Conference on Harmonization (ICH); or (b) an ICH Observer, being the European Free Trade Association (EFTA), as represented by Swissmedic and Health Canada; or (c) a regulatory authority associated with an ICH member through a legally-binding mutual recognition agreement including Australia, Iceland, Liechtenstein and Norway [10].

Ipca was identified through its FDA Drug Master File (DMF) and interviewed as the main manufacturer of primaquine API, the critical component which other pharmaceutical companies use to manufacture primaquine tablets (according to the FDA DMF [11]). A DMF is a submission of required information to the FDA that permits the FDA to confidentially review information about facilities, processes, or articles used in the manufacturing, processing, packing and storage of drugs [11].

### In-country perspectives

To assess demand and understand in-country concerns around SLD primaquine use, five countries in sub-Saharan Africa were chosen as case studies: Ethiopia, Senegal, Swaziland, Zambia, and Tanzania (Zanzibar). These countries were selected based on their expressed interest in using SLD primaquine, their goals of national or sub-national malaria elimination, their geographic diversity within Africa, and their varying population sizes. Key informants were purposefully selected for interview to represent the views of a range of levels and roles within the health system. A total of 43 in-country key informants were interviewed (Table 1, interview guide in Additional file 1), including but not limited to: 1) members of the National Malaria Control Programme (NMCP); 2) the local equivalent of a Drug Regulatory Authority; 3) key end users or implementers such as clinicians and professors with malaria expertise; and 4) in-country malaria partners (e.g., WHO, UNICEF, President’s Malaria Initiative). Additionally, 16 semi-structured interviews were conducted with global key informants with no specific country affiliation (interview guide in Additional file 1). Global key informants were comprised of academic malariologists, as well as organizations implementing research projects using SLD primaquine across multiple countries (Table 1). All individuals chosen for interviews were selected based on their professional affiliation, as well as relevant experience and knowledge in malaria.

**Table 1:** Number of key informant interviews conducted by country

Country	NMCP	Drug Regulatory Authority	Key end users or implementers	In-country partners	Total
Swaziland	4	0	0	1	5
Zambia	1	1	1	2	5
Senegal	4	3	3	4	14
Ethiopia	1	2	1	8	12
Zanzibar	4	3	0	0	7
TOTAL	14	9	4	16	43

### Data collection

Interviews with select manufacturers and key informants in sub-Saharan Africa were conducted from April to July, 2013 by one of the investigators (MN) to ensure consistency across interviews. Interviews followed a semi-structured interview guide and were conducted by phone, email, or during scheduled in-country meetings. Interviews with manufacturers explored the production landscape of primaquine and market demand for SLD primaquine. These interviews revealed a need to develop a lower strength tablet of primaquine, after which a second interview was conducted with Sanofi, selected purposively due to their experience with developing high quality, SRA-approved products, including various drug formulations. This second interview with Sanofi focused on establishing the process required to develop new tablets and/or paediatric formulations, and gauging manufacturer interest in undertaking these processes. The key informant interviews with in-country informants explored in-country perspectives of SLD primaquine use, including barriers to uptake, deployment options, and the feasibility of overcoming the barriers identified. Key informant interviews with global malaria researchers/implementers were focused on primaquine implementation, including supply, context for use, deployment options, and the feasibility of overcoming barriers to delivery. The majority of interviews were conducted in English with the exception of Senegal where, when necessary, the assistance of a French translator was requested to facilitate discussions. All interview questions were open ended, and handwritten or electronic notes were collected by the moderator (MN) during interviews.

### Analyses

Thematic analysis, a method that applies inductive reasoning by which themes emerge from the data through careful examination [12] was performed after familiarization of the data. The first level of analysis was conducted by an investigator (MN) who reviewed and analysed all documents, meetings, interviews, and email correspondences. A second level of analysis was performed by another investigator (RS). Together, MN and RS identified key themes based on frequency of appearance and contextual importance centered on the available literature, the primary research question, and consultation with topic experts. Emergent themes from interviews were discussed and compared regularly among authors (MN, LR, RG, and JH) to identify core consistencies across interviews and themes were collated to provide a view of barriers to SLD primaquine rollout and use. Recommendations were then synthesized for the barriers identified.

## Results

### Primaquine supply

Of the 51 manufacturers of primaquine identified, most were located in Asia. Sanofi and Remedica were the only producers of SRA-approved primaquine, and Ipca was identified as the main manufacturer of primaquine API. A few manufacturers of non SRA-approved primaquine were also interviewed.

Sanofi’s current primary customer is the United States Army, which places a single order of primaquine, usually on a yearly basis. Sanofi primaquine remains relatively expensive, with a current selling price of approximately $0.50 per 15 mg tablet as compared to Remedica tablets, which are approximately $0.03 per 7.5 mg tablet [9]. The high price is driven primarily by low demand and high quality of FDA-approved API. Sanofi FDA-approved primaquine is currently manufactured in Canada. A Sanofi plant in Colombia, GMP-certified by Colombian and Argentinean regulatory authorities, also produces 15 mg primaquine tablets, for regional markets, with greater capacity. Sanofi is working on optimizing production costs at both plants, and exploring various scenarios to register primaquine in malaria-endemic countries in which *P. falciparum* elimination could be considered.

Remedica tablets are SRA-approved by the Cyprus Ministry of Health-pharmaceutical services, a National competent authority listed by the SRA European Medicines Agency (EMA). Since 2011, Remedica has been the primary supplier of primaquine purchased through the GFATM, of which procurement has been growing rapidly over the past few years. In 2012, Remedica supplied approximately 34 million primaquine tablets to the GFATM [9], mostly for the radical cure of *P. vivax* malaria in southern and Southeast Asia. As SRA approval is often the minimum requirement for countries to procure drugs with international donor funding, Remedica supplies primaquine directly to countries, and also through donors, including UNICEF and the WHO/Roll Back Malaria Partnership.

Ipca is a major pharmaceutical manufacturer in India, producing approximately 2.4 million kg of assorted APIs per year, including approximately 48,000 kg of primaquine annually in two batches. Prices of primaquine phosphate API have increased over the past few years but this may change soon, as the US-based company Apicore LLC received an FDA Drug Master File for primaquine in July 2013, bringing more competition to the market.

Manufacturers that produce non SRA-approved primaquine stated a number of reasons for not seeking SRA approval for this drug. The most common reason was uncertainty around the commercial demand of an SRA-approved primaquine tablet. However, when told about growing interests in using SLD primaquine as a gametocytocide in African countries, manufacturers generally expressed an interest in developing a higher quality primaquine product and potentially seeking SRA approval or WHO prequalification. WHO prequalification is of particular interest for SLD primaquine, as most anti-malarials to date, including ACT, have undergone this route for use in sub-Saharan Africa. However, it is currently not possible for SLD primaquine to proceed through WHO prequalification, as SLD primaquine would first need to be listed on the WHO prequalification programme Expression of Interest (EOI) list, after which the WHO prequalification programme would be willing to accept a dossier for SLD primaquine.

### Dosing

In-country interviews with key end users revealed that a major barrier to primaquine uptake is the lack of clarity of primaquine dosing in clinical settings. The current WHO recommendation is a weight-based 0.25 mg/kg dose, which is challenging to implement in actual field settings, where dosing is usually based on patient age. Age-based dosing bands are derived from the therapeutic dose range, a weight-based range spanning from the lowest efficacious dose to the highest safe dose, translated using weight-for-age data [13]. As primaquine safety depends on dose, there is a critical need to inform dosing practices by defining the therapeutic dose range of SLD primaquine for *P. falciparum* gametocytes.

**Fig. 1:**
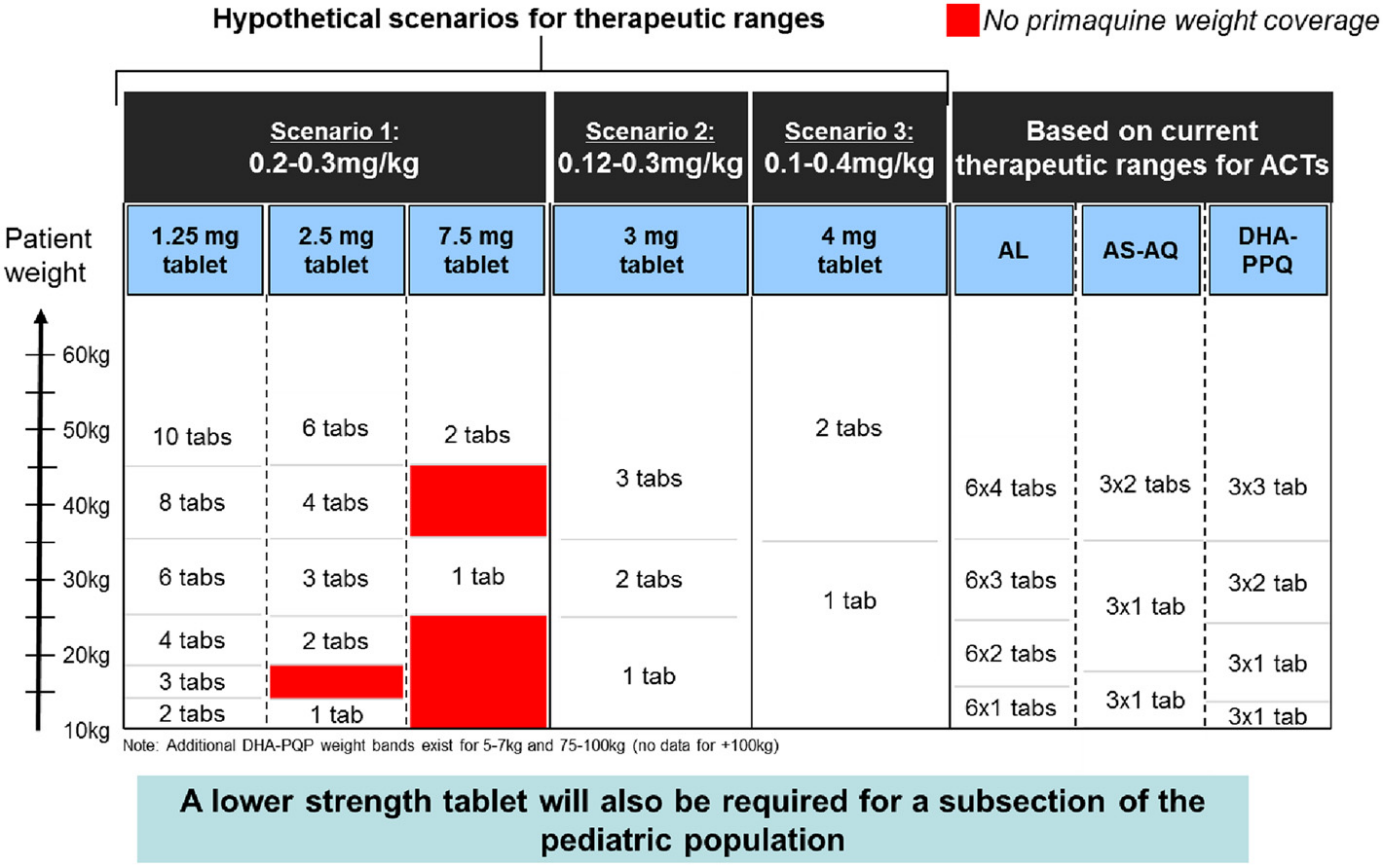
The effects of hypothetical primaquine therapeutic dose ranges on dosing schedule

The width of the therapeutic dose range of primaquine also has implications for dosing practices in clinical settings. Figure 1 illustrates several hypothetical therapeutic dose ranges of primaquine, and how these ranges may affect dosing practices, per the WHO recommendation. For example, if a 10 kg and a 20 kg child are dosed, and the therapeutic dose range is defined as 0.12 to 0.3 mg/kg, both children could be treated with a single 3 mg tablet of primaquine. However, if the therapeutic dose range were narrower, the dosing regimen becomes more complicated; the 10 kg child would require one 2.5 mg tablet while the 20 kg child would need two 2.5 mg tablets. Tablet strength becomes important, as bigger strengths may not afford weight-based coverage for paediatric patients, as highlighted in red (Fig. 1).

As the WHO recommends that SLD primaquine be used with an ACT, the dosing bands for commonly used types of ACT are also shown in Fig. 1. Dosing practices would be easier if the dosing bands for ACT and primaquine could be aligned, but this will unfortunately be challenging, as the dosing bands for artesunate-amodiaquine (AS-AQ) differ significantly from those for artemether–lumefantrine (AL) and dihydroartemisinin–piperaquine (DHA–PPQ).

To enable the use of SLD primaquine across the paediatric population, researchers and manufacturers agreed on a need for the development of a lower strength tablet for SRA approval or WHO prequalification. The current SRA-approved tablets, available from Remedica and Sanofi, are only available in 7.5 or 15 mg strengths, respectively, which do not allow for accurate dosing in sub-sections of the paediatric population. Although generic companies in India produce primaquine tablets at 2.5 mg strength, these are not SRA-approved and therefore do not meet the quality standards set out by international donors, and the therapeutic dose range is needed to determine whether 2.5 mg is a suitable tablet strength for SLD primaquine. Current SRA-approved primaquine tablets also do not have “splitting lines” or “break lines” which help facilitate pill-splitting for the administration of lower doses. Even if these lines were added, however, the tablets are already too small in size to split accurately; the 7.5 mg Remedica tablet is only 6 mm in diameter.

Manufacturers expressed an interest in developing lower strength primaquine tablets, but due to the complexity of these processes, the exact tablet strength needed to dose paediatric populations first needs to be established. Usually, tablets are made smaller using the same ratio of excipients to API, known as a “homothetic mix.” However, manufacturers stated that the primaquine tablets currently available are too small to be further reduced. Since the ratio of API to excipients determines the absorption and bioavailability of the API, seeking regulatory approval for changes to this ratio usually requires the submission of bioequivalence study data, which manufacturers expressed would both extend the amount of time required for regulatory approval, and furthermore add complexity to the process. It is possible to expedite the regulatory approval process by applying for a biowaiver, and although the properties of primaquine are likely to fulfill these biowaiver requirements [14], manufacturers expressed a general lack of understanding of this process. In the event that biowaiver requirements cannot be fulfilled, and furthermore the absorption and bioavailability of the API for homothetic mix(es) are not adequate for regulatory requirements, liquid-based formulations would need to be developed to allow for the availability of lower strength doses of primaquine.

Manufacturers also noted that small tablets are difficult to administer to paediatric patients, and dispersible tablets or liquid formulations are usually preferred. However, they also stated that new formulations are expensive and time consuming to develop, and may present additional regulatory hurdles since bioequivalence data would likely be required. Manufacturers also mentioned that it is possible to crush currently available primaquine tablets for paediatric use. At the time of study, the efficacy and reproducibility of this method in clinical practice had not been tested, but Sanofi has since released a Standard Operating Procedure for this practice [15]. Manufacturers expressed however, that the crushing of tablets is not an optimal solution to paediatric dosing, as the primaquine API is extremely bitter and is frequently found not to be palatable. Thus, they suggested that a child-friendly formulation would be preferred, and should furthermore strive to mask the bitter taste of primaquine API.

### Country perspectives

African countries have little experience administering primaquine as part of routine malaria case management, and in-country interviews with key informants in National Malaria Control Programmes and their partner organizations revealed varying levels of understanding surrounding the use of primaquine. While some individuals were unaware that SLD primaquine could be used to clear mature *P. falciparum* gametocytes, others demonstrated a detailed understanding of primaquine as a gametocytocide. Despite varying degrees of knowledge, all countries in our case studies expressed interest in adopting primaquine as a transmission-blocking agent for *P. falciparum* malaria.

Respondents who were aware of primaquine and its use as an anti-malarial drug cited several benefits. Primarily, primaquine is an effective transmission-blocking tool for the control and elimination of *P. falciparum* malaria. In Ethiopia, the Ministry of Health (MoH) noted that the use of primaquine could help to achieve their ambitious goal of malaria elimination in low-transmission areas by 2015. In Senegal, where malaria transmission levels are higher and elimination is not the primary objective, experts from the NMCP agreed on the transmission-blocking potential of primaquine as a critical tool for reducing malaria transmission. In Zanzibar, health experts emphasized the need for primaquine to eliminate *P. falciparum* malaria.

### Safety

Despite interest in adopting primaquine as a *P. falciparum* gametocytocide, all countries expressed safety concerns of the haemolytic potential of primaquine in G6PDd individuals. To facilitate the adoption of the WHO recommendation and to inform local deployment strategies, health experts requested that maps of the local prevalence of G6PD deficiency be made available. A second concern noted by academics, drug regulators, international organizations and national programmes arises from the potential risks that primaquine may cause in vulnerable patient groups, including small children and patients with other co-morbidities including HIV, sickle cell anaemia or malnutrition. Doctors and members of the NMCP in Swaziland, a country with the highest HIV prevalence at 26.5% [16], voiced unease about the use of primaquine among HIV-positive individuals because haematologic disorders, including anaemia, are particularly common in HIV-positive patients [17].

### Regulatory issues

Countries explained a number of regulatory processes that may introduce delays and pose challenges to the adoption and implementation of SLD primaquine. Country anti-malarial drug guidelines are only reviewed, revised, and updated on a periodic basis potentially delaying anti-malarial policy changes at the national level. However, countries suggested the possibility of publishing a “circular” or interim recommendation, which can act as a standing recommendation until new guidelines are published. The process for policy change also varies between countries. In Zambia, a pre-requisite to drug registration is the inclusion of the product in existing malaria treatment policy guidelines whereas in Senegal, a drug is much more likely to be included in their guidelines if it is already registered for in-country use.

Regulatory requirements for primaquine registration presented additional obstacles and delays to the adoption of SLD primaquine as policy. The formal registration of drug products can take anywhere from one month, which is the case for Zanzibar, to up to two years, as is the case for Ethiopia and Zambia. Fortunately, most countries have an expedited review process, a process that generally takes anywhere between three to six months. In Ethiopia and Senegal, an expedited review is merited when the drug under consideration has demonstrated a clear public health need. In Zambia, an expedited review is possible if the drug is registered in another Southern African Development Community country. Swaziland does not currently have a drug regulatory authority, and generally follows South Africa’s recommendations.

Some countries also have additional regulations that pose further delays; in Senegal, manufacturers are required to submit product dossiers to make sure that products are safe and effective for use by local patients. Occasionally, in-country registration requires that the product be deemed as cost-effective.

### Field deployment

Country programmes, international organizations and researchers expressed differing opinions on the best methods with which to deploy primaquine. They described three dominant strategies for the use of primaquine to interrupt malaria transmission: the treatment of confirmed clinical cases only, mass screen and treat (MSaT) strategies, and mass drug administration (MDA) campaigns. MSaT strategies aim to screen entire populations of interest, treating all individuals with detectable levels of malaria parasitaemia. MDA entails the administration of a curative regimen of anti-malarials to all individuals within a given locale, regardless of parasitaemia or the presence of symptoms [18].

Among those interviewed, the most widely accepted SLD primaquine deployment option was the treatment of confirmed clinical cases, noted by key opinion leaders, researchers, local doctors, and members of the NMCPs. However, there was interest in broader deployment strategies for primaquine as its use for clinical cases might have limited impact on the overall parasite reservoir. Some international organizations, governmental agencies, and researchers advocated for MSaT; in Ethiopia, the MoH recommended the use of MSaT during regional epidemics and MDA if the malaria slide positivity rate exceeds 50%. The NMCP in Swaziland was also open to targeted MSaT with primaquine. At the time of study, researchers in Zambia were considering the addition of primaquine to an MSaT study using ACT. Malaria researchers expressed a number of drawbacks to MSaT however, including operational challenges and high costs.

Other malaria researchers believed that MDA could best ensure malaria elimination within a population. However, the notion of deploying primaquine in an MDA campaign raised numerous concerns of safety in individuals with G6PD deficiency. Experts raised ethical questions about MDA, as to whether it is acceptable to pose potential risks to individuals in order to benefit entire populations. Furthermore, country programmes were hesitant to implement a strategy that is not currently recommended by the WHO.

## Discussion

Supply and demand are critical considerations for growing interest in the use of SLD primaquine to block *P. falciparum* transmission. This is the first study to identify key barriers to the large-scale rollout of SLD primaquine in sub-Saharan Africa, from which the following recommendations for researchers, manufacturers, and malaria programme managers can be derived.

Malaria programmes and manufacturers are in need of a defined therapeutic dose range for SLD primaquine. Not only will the therapeutic dose range inform the development of new, lower strength primaquine tablets and/or formulations by manufacturers; this dose range will also guide therapeutic dosing regimens in the field, and allay safety concerns of haemolytic effects in G6PDd individuals, as the upper bound of the therapeutic dose range is the highest safe dose in G6PDd individuals.

Manufacturers and malaria programmes agree on a need for new, lower strength primaquine tablets to ensure accurate dosing among paediatric subsections of the population. After the therapeutic dose range of SLD primaquine is established, manufacturers will gauge the level of investment to undertake for the development of a new primaquine product. A less expensive approach would be to develop a new, lower strength primaquine tablet, including the possibility of applying for a biowaiver. Larger investments would be needed to develop new formulation(s) of primaquine, including a paediatric formulation that masks the bitter taste of the primaquine API. As manufacturer decisions are based on the profitability of the market and level of investment required, the demand for new primaquine product(s), once available, should be periodically forecasted, both to ensure that production levels are sufficient, and to assess the size of the potentially growing market for SLD primaquine products.

Defining the therapeutic dose range will likely be the first step toward allaying safety concerns expressed by malaria programme officers. Further data will be needed to define the risk profile of SLD primaquine in vulnerable populations, notably G6PDd individuals, small children, those with malnutrition, anaemia, HIV, and/or tuberculosis, with and without treatment for these comorbidities. The safety profile in these populations can either be established through clinical trials, or be studied through pharmacovigilance of programmes that are already using primaquine as per the WHO recommendation. Pharmacovigilance databases need to collect information on birth outcomes in women who take primaquine before they are aware of their pregnancy, as prescribing instructions warn against the use of primaquine in pregnancy and breastfeeding due to a lack of data.

Malaria programme managers also expressed an interest in G6PD prevalence maps, to better assess local haemolytic risks in G6PDd populations. Currently available G6PD prevalence maps are based on variant proportions or allele frequencies that have been derived through population-level screening surveys, suggesting that in Africa, G6PD deficiency is common, with a prevalence of up to 32% in certain regions, mostly comprised of the mildly primaquine-sensitive A- variant of G6PD deficiency [19]. Importantly, G6PD prevalence maps are limited by the quality of screening methods employed, and prevalence maps are unlikely to be useful to malaria programme managers unless they achieve very high resolutions [19]. It will be most important to identify variants of G6PD deficiency that are highly sensitive to the adverse effects of primaquine, and to then consider the combination of frequency and severity. While frequent mild variants may not be an issue, rare severe variants might be problematic, and in areas where severe variants are common, SLD primaquine should be used with caution until its safety is established.

There are two methods to screen for G6PD activity levels in order to establish prevalence maps, both of which have important implications on their reliability. The first method is to conduct genetic testing, which can provide a clear diagnosis of a specific gene mutant, but is limited by its inability to recognize unknown variants of G6PD deficiency [20]. A second method, which is the most widely used method, is to conduct biochemical, phenotypic testing of G6PD enzyme activity to provide a quantitative or qualitative readout, depending on the level of infrastructure available [20]. However, phenotypic assays are limited by their ability to detect heterozygous females, as the G6PD gene is X-linked, and lyonization, or partial inactivation of one female X chromosome, result in wide variations in phenotypic G6PD activity that can lead to the misclassification of G6PDd women as G6PD normal. Biochemical approaches are also affected by the duration of malaria infection, anaemia of the patient, and other haematological parameters, further increasing the chances of misclassifying a G6PDd person as G6PD normal. Importantly, both genetic and biochemical methods require advanced laboratory infrastructure and skilled technicians [21]. Recently, the BinaxNow® and CareStart® point-of-care G6PD tests became available. While point-of-care tests do not require laboratory infrastructure, they otherwise carry the same limitations posed by qualitative, phenotypic testing. The generation of reliable, high-resolution G6PD prevalence maps will be labor intensive and costly, and is furthermore challenged by a lack of consensus on the cut-off level of enzymatic activity required to safeguard against severe haemolysis. Therefore, the generation of prevalence maps must be accompanied by clinical studies and/or pharmacovigilance activities to ensure that the method of G6PD testing employed, given its limitations, can sufficiently denote the risks of severe haemolysis to in-country programme managers.

The in-country procurement of primaquine also faces numerous regulatory challenges, and suppliers need to ensure that SLD primaquine products are either WHO prequalified and/or SRA-approved. These regulatory requirements will allow for the drug to be procured through GFATM and other international donor funding channels, and likely enable expedited in-country registration. Currently, no primaquine products have obtained WHO prequalification, revealing a need for primaquine tablets to be placed on the WHO prequalification EOI list. As primaquine is recommended for use in WHO treatment guidelines, this process is expected to be straightforward [22]. The priority SLD primaquine product that should be placed on the WHO EOI list for prequalification will be the optimal tablet strength, based on the therapeutic dose range, as soon as it is determined. This will enable for the SLD primaquine product to be available to countries. In the meantime, it would also be helpful for suppliers of non SRA-approved primaquine products to place currently available tablet strengths on the WHO prequalification EOI list. This would increase the number of suppliers that can provide primaquine through donor funding mechanisms, since the Sanofi (15 mg) and Remedica (7.5 mg) products are currently the only two available products that are procurable through donor funding. If primaquine products are placed on the WHO prequalification EOI list, manufacturers will be aware that a market exists, which can encourage investments in the further development of SLD primaquine products, as well as the regulatory approvals needed.

While SRA approval and/or WHO prequalification are often helpful for in-country registration, other regulatory requirements vary at a country level. Countries considering the use of SLD primaquine should explore country specific requirements ahead of time, in particular, whether methods to expedite in-country registration are available.

Finally, the optimal delivery strategy for SLD primaquine is at present unclear. Although the most widely accepted use of primaquine is through the treatment of clinical cases as per the WHO recommendation, there is interest in the broader use of SLD primaquine through MSaT or MDA campaigns. Recent studies in Burkina Faso and Zanzibar have shown that MSaT using AL alone did not reduce the incidence of *P. falciparum* malaria in either location [23, 24]. It is unclear whether effectiveness could be improved through the addition of SLD primaquine, or whether MSaT strategies themselves are limited by the sensitivity of malaria RDTs [25]. In any case, given the high cost of screening, MDA strategies are likely to be much less costly than MSaT. To date, there is no concrete evidence that the use of SLD primaquine on a large-scale would impact *P. falciparum* transmission in sub-Saharan Africa, largely due to challenges with measuring malaria transmission at a population level. Mathematical malaria transmission models offer a useful resource, suggesting that with high levels of coverage, the addition of SLD primaquine to an artemisinin–piperaquine MDA deployment strategy provided *P. falciparum* transmission-blocking effects, both accelerating malaria elimination strategies and reducing the rate of emergence of artemisinin-resistant malaria parasites [26]. The use of primaquine through an MDA strategy is indeed controversial, hinging on risk-benefit ratios and safety, as primaquine is the only drug available to stop malaria transmission from the human to the mosquito [7, 27].

### Limitations

As this exercise focused on garnering an understanding of the current manufacturing, regulatory and demand landscape for SLD primaquine use, key manufacturers of primaquine and a small group of purposefully selected key informants from five African countries were chosen for interviews. Inference is therefore limited and the results may not be generalizable to other contexts. Nonetheless, we attempted to conduct a diverse array of interviews with representatives from industry and in-country key informants, to capture the current perspectives surrounding the production landscape for primaquine and in-country perspectives on the use of primaquine as a *P. falciparum* gametocytocide in sub-Saharan Africa.

## Conclusion

Demand for SLD primaquine is growing rapidly, yet multiple barriers to SLD primaquine rollout exist. Research is needed to define the therapeutic dose range, to develop a paediatric formulation, and to establish interactions with co-morbidities and treatments for HIV/tuberculosis. Regulatory processes should be sought early; countries should explore regulatory requirements ahead of time, and there is an urgent need for suitable strength primaquine tablets to obtain WHO prequalification. It is critical to address these barriers to support the WHO recommendation, particularly as programmes consider broadening the use of SLD primaquine through MSaT or MDA strategies.

## Additional file

Additional file 1: An assessment of the supply, programmatic use, and regulatory issues of single low-dose primaquine as a Plasmodium falciparum gametocytocide for sub-Saharan Africa: Interview Guides. Interview guides used for semistructured interviews with suppliers, in-country key informants, and research experts implementing primaquine programmes.

## Abbreviations

ACT: Artemisinin-based combination therapy
AL: Artemether–lumefantrine
API: Active pharmaceutical ingredient
AS-AQ: Artesunate–amodiaquine
DHA–PPQ: Dihydroartemisinin–piperaquine
DMF: Drug Master File
EFTA: European Free Trade Association
EMA: European Medicines Agency
EOI: Expression of Interest
FDA: Food and Drug Administration
GFATM: The Global Fund to fight AIDS, Tuberculosis, and Malaria
G6PD: Glucose-6-phosphate dehydrogenase
G6PDd: Glucose-6-phosphate dehydrogenase deficiency
ICH: International Conference on Harmonization
MDA: Mass drug administration
MSaT: Mass screen and treat
NMCP: National Malaria Control Programme
RDT: Rapid diagnostic test
SLD: Single low-dose
SRA: Stringent Regulatory Authority
WHO: World Health Organization
