# Supplementary material for: An assessment of the supply, programmatic use, and regulatory issues of single low-dose primaquine as a Plasmodium falciparum gametocytocide for sub-Saharan Africa
Source: Malar J. 2015 May 15;14:204. doi: 10.1186/s12936-015-0714-3 (PMC4446184; doi:10.1186/s12936-015-0714-3)
Supplement: Additional file 1: — An assessment of the supply, programmatic use, and regulatory issues of single low-dose primaquine as a Plasmodium falciparum gametocytocide for sub-Saharan Africa: Interview Guides. Interview guides used for semistructured interviews with suppliers, in-country key informants, and research experts implementing primaquine programmes. [file 12936_2015_714_MOESM1_ESM.pdf]

An assessment of the supply, programmatic use, and regulatory issues of single low-dose primaquine as a *Plasmodium falciparum* gametocytocide for sub-Saharan Africa: Interview guides

Ingrid Chen*, Eugenie Poirot*, Mark Newman, Deepika Kandula, Renee Shah,
Jimee Hwang, Justin Cohen, Roly Gosling, Luke Rooney
(*These authors made equal contributions to this manuscript)

Contents:

1. Interview guide for suppliers: pages 2 – 3
2. Interview guide for in-country interviews: pages 4 – 6
3. Interview guide for research experts implementing primaquine projects and/or programs: pages 7 – 8

All interviews were introduced with the following information:

- Recent change in WHO guidelines for the usage of primaquine
- Interest to better understand primaquine's potential to drive towards elimination (project funded by Gates)
- Global Health group performing two clinical trials to better understand the product
- Clinton Healthcare Access Initiative assessing both the supply of the product as well as the demand (and what barriers would exist to utilizing the product in-country for range of strategies from usage in confirmed patients to mass screen and treat to MDA)

1. Interview Guide for suppliers

Current State

- What dosages of PQ do you produce?
- Are there pediatric formulations of PQ?
- Can you crush and / or split the PQ tablets (e.g., if you produced 10mg, could you split it into 5mg tablets)?
- Do you manufacturer primaquine API? If not, where do you source it from and have there been any issues with supply or quality?
- Can you provide us with an estimate of the quantities of PQ you produce (by dosage)? What is your maximum production capacity across these dosages? Is the factory / production dedicated to PQ? If not, what other products are produced in the factory?
- Where is your primary (and secondary) market for PQ, in terms of both countries and buyers?
- In which countries is your product registered?
- Who are your primary competitors for PQ?
- Does the plant where PQ is produced have GMP certification? If so, from whom?
- Does the plant where PQ is produced also produce ACTs? If so, which ACTs do you produce? Are these drugs packaged in the same facility?
- Have there been any manufacturing issues at the plant? If so, are these regular occurrences from manufacturing the drug?
- Are there significant demand fluctuations?
- Do you experience out-of-stocks?
- Can you provide us with an estimate of the cost and price associated with the production of PQ?

Transition

- As mentioned initially, we are talking with malaria experts in country assessing the future potential market
- Country interest: based on our initial discussions, there is a significant amount of interest in the use of PQ, but a concern for the supply of SRA approved or WHO prequalified PQ
- Market size: based on our initial market sizing exercise, there is a wide range in terms of the market size because of the different deployment strategies and countries that might procure the drug

Future State

- Is this a market you might be interested in? Are there specific make or break factors for participating in this future market for you?
- Do you foresee future fluctuations in demand? What drives these fluctuations?
- Can you produce different (~2.5mg) dosages of PQ? If so, what would be the additional cost and where would you seek approval of this new dosage?
- Could you package PQ in the same tabs as your ACT treatment? What would be the impact of this manufacturing change on manufacturing costs? Supply chain? Distribution?

- If PQ was supplied to Africa, what would be the impact on your supply chain costs?
- What do you estimate to be the additional cost to produce PQ in a GMP certified facility in terms of upfront investments and additional run rate cost?
- If all or some combination of these changes were made, would you expect there to be an impact on supply reliability? Interest in the market?

Interview Guide for in-country interviews

Questions for Primaquine Usage:

- Brief project description touching upon the:
  - Recent change in WHO guidelines for the usage of primaquine
  - Interest to better understand primaquine's potential to drive towards elimination
  - Global Health Group performing two clinical trials to better understand the product
  - Clinton Healthcare Access Initiative (CHAI) assessing both the supply of the product as well as the demand (and what barriers would exist to utilizing the product in-country for range of strategies from usage in confirmed patients to mass screen and treat to mass drug administration (MDA))
- How aware of primaquine are you? Is primaquine registered in country and / or included in the country guidelines?
  - If no, discuss barriers to uptake
  - If yes:
    - What is primaquine registered / in the guidelines for?
    - Who are the manufacturers / suppliers of the product? Would you be willing to put us in touch with them?
    - What are the dosages of the product?
    - When was primaquine incorporated into the guidelines? Has it ever been ordered or procured?
    - Are there any issues with the supply of the product?
    - Are there any specific concerns on the usage of the drug? How are these concerns mitigated / managed?

Questions for Current Barriers to Uptake:

- Registration
  - What is the registration process within the country for a new product? What are the key components of this process?
  - Who is involved in the decision making process?
  - How long does the registration process typically take?
  - Are there specific components of the registration process that suppliers / manufacturers find particularly difficult?
  - Have suppliers attempted to register primaquine?
  - If so, what was the result and reasoning behind the decision?
  - If primaquine was rejected, what would need to occur for primaquine to be registered?
- Guidelines
  - Is primaquine included in the guidelines? If so, are there any specific concerns? If not, is there a specific reason why not?
    - Awareness?
    - Clinical concerns (efficacy, indications, etc.)?
    - Cost concerns?

        - Administration concerns?
        - Product availability concerns?
    - What is the impact/necessity of national treatment guidelines on public and private sector uptake of new products?
    - What is the process by which the treatment guidelines are revised?
        - Is there a technical working group and if so, who are the participants?
        - How often do the treatment guidelines get revised? When are they next due to be revised?
        - What is the normal lag time between revised WHO recommendations and updated treatment guidelines?
        - Does the country need to run its own studies or conduct pilots before inclusion of new products in the guidelines?
    - What about guidelines dissemination? How long does it usually take to reach the facility level? What types of dissemination strategies are used – e.g. is health worker training conducted on new/revised guidelines?
    - Is primaquine included on the essential medicines lists?
        - How are these lists revised?
        - What would need to occur for primaquine to be included on the list?
        - What is the bearing of these lists on the uptake of new products?
- Procurement / Tendering
    - What are the gaps between guidelines adoption and central level procurement?
    - Where does funding come from for malaria medication? Are there restrictions on the types of products that can be procured with this funding?
    - What is the ratio of public sector v. private sector procurement and case load?
    - For the public sector, how are final buying decisions made? Which government and non-government partners play a role in the process?
    - Are in-country buyers/procurement agents aware of primaquine?
    - If so, are there specific reasons they are or are not bought?
- Supply Chain
    - How does the distribution of malaria products work between the central, regional, and primary levels?
    - How many supply chains are there?
    - How autonomous are the regions in terms of product selection/ordering decisions?
    - How are newly available products communicated to the facility level?
- Facility Level
    - Which types of facilities treat malaria, both in the private and public sectors?
    - Who at the facilities is responsible for placing orders/determining what to order?
    - Are the individuals at the facilities aware of primaquine?
    - Are there concerns with the product at the facility level?
    - Has any training been conducted for the product? Would the staff need to be retrained for the product?

Questions for Primaquine Deployment Strategies:

- As mentioned, looking at a range of strategies for the usage of primaquine in confirmed patients to mass screen and treat to MDA; what would be the impact of these different deployment strategies on the barriers discussed above?
- Would you have a preference for a specific approach? Why?
- Do you have any concerns on any of the approaches?
- Are there any other approaches you think may make sense?

Questions for Potential to Address Barriers:

- Does there seem to be any traction within the public/private sectors for accelerating uptake of primaquine?
- What are the current strategies and do they adequately address the observed barriers?
- What are strategies employed for the usage of other drugs that worked effectively?
- What are the timelines/indicators for these strategies?
- Which non-government partners are active in the malaria space and what role do they play?
- Do government and non-government partners have the capacity needed to carry out the plan?
- What would uptake realistically look like in the short-term, medium-term, long-term?

3. Interview Guide for research experts implementing primaquine projects and/or programs

PQ Implementation:

- Can you talk to us about the work that you have done with PQ?
- How are you looking to implement PQ? What do you want the policies for PQ to be?
- Do you think the same format should be followed in other countries? If not, what should be changed?
- Do you provide ACT in conjunction with PQ?
- What is your experience with the adverse side effects?
- Would you dose patients with PQ who are known to have G6PDd?
- Were there any barriers / constraints that needed to be overcome to use PQ?
- What are the characteristics of the areas that you include in your work? What is the level of endemicity in these areas?
- Based on your work, what was the impact of PQ on malaria endemicity?

Supply of PQ:

- Who do you source PQ from?
- Where there multiple options for procuring PQ?
- What is the volume that you procure?
- What are the PQ treatment weight bands that you follow? What dosages do you provide to patients?
- Can you crush and / or split the PQ tablets (e.g., if you produced 10mg, could you split it into 5mg tablets)?
- How reliable is the procurement of PQ?
- What is the lead time?
- Do you know if the plant has GMP certification?
- Can you provide us with a cost estimate of procuring PQ?

Speculation on Project:

- Do you think it would be beneficial to include PQ in malaria treatment guidelines? Why?
- What type of deployment strategy do you think is best for PQ? Why?
- Do you have any concerns on any of the approaches?
- Are there any other approaches you think may make sense?

Questions for Potential to Address Barriers:

- Does there seem to be any traction within the public/private sectors for accelerating uptake of primaquine?
- What are the current strategies and do they adequately address the observed barriers?
- What are strategies employed for the usage of other drugs that worked effectively?

- What are the timelines/indicators for these strategies?
- Which non-government partners are active in the malaria space and what role do they play?
- Do government and non-government partners have the capacity needed to carry out the plan?
- What would uptake realistically look like in the short-term, medium-term, long-term?
